# Racial and Gender Differences in the Presentation of Pruritus

**DOI:** 10.3390/medicines6040098

**Published:** 2019-09-27

**Authors:** Katherine A. Whang, Raveena Khanna, Jamael Thomas, Crystal Aguh, Shawn G. Kwatra

**Affiliations:** 1 Department of Dermatology, Johns Hopkins University School of Medicine, Baltimore, MD 21231, USA; kwhang5@jhmi.edu (K.A.W.); rkhanna8@jhmi.edu (R.K.); jamael.thomas@utsouthwestern.edu (J.T.); cagi1@jhmi.edu (C.A.); 2 School of Medicine, University of Texas Southwestern Medical Center, Dallas, TX 75390, USA; 3 Johns Hopkins Bloomberg School of Public Health, Baltimore, MD 21231, USA

**Keywords:** pruritus, itch, prurigo, nodularis, atopic, dermatitis, race, gender, comorbidities, demographics

## Abstract

**Background:** Pruritus is a common disease symptom with a variety of etiologies known to reduce patient quality of life. We aimed to characterize the racial and gender differences in the presentation of pruritus for itch-related patient visits both within a single institution and nationally. **Methods:** Cross sectional study of patients ≥ 18 years old seen at Johns Hopkins Health System between 1/1/12 and 1/1/18. Results were compared to data from 2005–2011 from the National Ambulatory Medical Care Survey (NAMCS) and the National Health Ambulatory Medical Care Survey (NHAMCS). **Results:** Our findings indicate that itch patients at JHHS (*n* = 18,753) were more likely to be black compared to white patients (37% vs. 19%, *p* < 0.01) when compared to patients without itch—a trend also noted nationally based on data from NAMCS/NHAMCS (26% vs. 21%, *p* = 0.05). Black itch patients are also more likely to be diagnosed with prurigo nodularis (OR 2.37, *p* < 0.0001), lichen planus (OR 1.22, *p* < 0.0001), and atopic dermatitis OR 1.51, *p* < 0.0001). Female itch patients are more likely to be diagnosed with autoimmune (OR 1.66, *p* < 0.0001) and psychiatric comorbidities (OR 1.2–1.8, *p* < 0.0001) than male itch patients. When compared to black itch patients nationally, white itch patients were more likely to visit a dermatologist (29% vs. 18%, *p* = 0.028). Our data can identify associated conditions and demographic differences but are unable to support a causal relationship. **Conclusions:** Black and female patients are more likely to present with pruritus, a symptom associated with comorbidities such as prurigo nodularis, lichen planus, atopic dermatitis, and psychiatric conditions.

## 1. Introduction

Pruritus is a common disease symptom known to negatively impact patient quality of life, contributing to both anxiety and depression. As a pervasive symptom, itch is present in an estimated 1% of all outpatient visits in the United States, occurring at any age and associated with a variety of dermatologic, systemic, neurologic, and psychiatric etiologies [1].

Pruritus arises from several different etiologies, from dermatoses and inflammation to infection, metabolic disease, cancer, psychiatric disease, drug application, and more. While the exact mechanism of pruritus is not fully understood, current evidence reveals the complex interplay between the skin, peripheral itch mediators such as histamine, neuropeptides, prostaglandins, and serotonin, and receptors, and the central nervous system. Sensation of itch, along with burning pain and heat, are transmitted through slow-conducting unmyelinated C-fibers. This pruritic information is thought to travel to the spinal cord via the dorsal root ganglion and travel along the spinothalamic tract [2]. Although histamine mediates a major itch pathway, non-histaminergic itch can be resistant to anti-histamine treatment and presents a challenge to clinicians.

Various different itch pathways have been implicated in different pruritic diseases. Atopic dermatitis (AD) is one example of a particularly pruritic dermatologic condition and is characterized by systemic epidermal barrier dysfunction. Increased transepidermal water loss, as commonly seen in AD, is highly associated with itch intensity [3]. Furthermore, the epidermal barrier dysfunction contributes to the entrance of irritants and pruritogens in AD patients, contributing to high pruritus burden. In contrast, psoriasis, which has been shown to have a lower burden of itch, is thought to be mediated by neurogenic inflammation and abnormal expression of neuropeptides such as substance P, calcitonin gene-related peptide, and somatostatin [4]. Studies have demonstrated that pruritus burden in psoriasis is associated with reduced health-related quality of life [5]. In terms of systemic causes of itch, pruritus due to cholestatic liver disease is believed to be due to pruritogens such as bile acids, lysophosphatidic acid, opiates, and progesterone derivatives [6].

Although gender and race are important factors in understanding disease etiology, there is limited knowledge on how these differences affect the presentation of pruritus. A 2013 study found that patients seen in visits for itch were more likely to be black or Asian than patients seen for other reasons [1]. Additionally, a German-based population study showed that women had higher visual analogue scale scores for itch severity, reported higher impact on quality of life, and presented with chronic scratch lesions more often than men, indicating gender-specific differences in underlying disease and burden among patients with chronic pruritus [7].

In our study, we sought to further understand the gender and racial differences in the presentation of pruritus by assessing itch-related patient visits in a tertiary care health system and national datasets.

## 2. Methods

Electronic health record system EPIC was used to collect anonymous aggregate-level retrospective data on patient demographics and comorbidities. We recorded the total number of patients age 18 years and older who had a chief complaint of “Pruritus” or a diagnosis of “Itching of the skin” from 1/1/12 to 1/1/18 using the systemized nomenclature of medicine—clinical terms (SNOMED-CT). Of these patients, we determined the percentage of patients with various dermatologic and non-dermatologic conditions known to be associated with pruritus and further stratified our results based on gender and race. Patient visits reporting itch were compared to controls, over the same time frame, of visits at JHHS that were not for itch. Odds ratios (ORs) were calculated to evaluate the strength of association of itch visits and diagnosis of various conditions when compared to the general patient population. Statistical significance was set at *p* < 0.05.

We also used data from the National Ambulatory Medical Care Survey (NAMCS) and the National Health Ambulatory Medical Care Survey (NHAMCS), nationally representative surveys conducted by the Centers for Disease Control and Prevention surveying office-based physicians, hospital emergency departments, and outpatient centers. Data from 2005–2011 were compiled to include entries for “Skin itching” in the “Reason for visit” field based on international classification of diseases—ninth revision, clinical modification (ICD-9-CM). The provided patient visit weights were applied to adjust for sampling probability and clustering effects and to determine nationally representative estimates of outpatient visits. Demographic and visit characteristics were compared between itch-related and non-itch-associated patient visits, for which a Pearson’s chi-squared test was calculated to determine statistical significance. When adjusting for potential confounders such as age, sex, socioeconomic status, and region, an OR was generated by performing multivariable analysis using logistic regression.

## 3. Results

Table 1 shows the characteristics of visits for itch at JHHS and nationally, based on data from NAMCS/NHAMCS. Of the 18,753 patients seen for itch at JHHS, a greater percentage were female when compared to patients seen for other reasons (66% vs. 54%, *p* = 0.01). Patients seen for itch-related concerns were also more likely to be black (37% vs. 19%, *p* < 0.01) and Hispanic/Latino (5.8% vs. 2.7%, *p* = 0.05) when compared to patients without itch at JHHS. According to the national datasets, while a greater proportion of patients seen for pruritus were black (26% vs. 21%, *p * = 0.05), there were no other statistically significant differences in demographic characteristics in patients seen for itch complaints when compared to those without itch.

**Figure 1:**
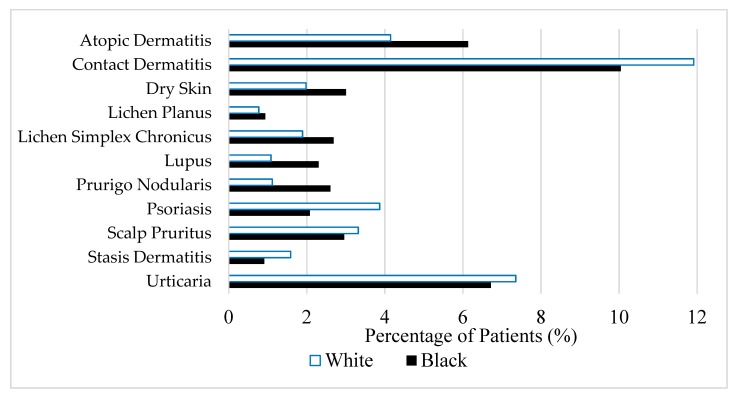
Percentage of white and black patients seen for itch diagnosed with various conditions.

The data from JHHS were further utilized to assess racial differences in the percentage of itch patients who presented with various conditions commonly associated with pruritus symptoms. Of the patients seen for itch, blacks were more likely than whites to be diagnosed with atopic dermatitis (OR 1.51, *p* < 0.0001), lichen planus (OR 1.22, *p* < 0.0001), prurigo nodularis (OR 2.37, *p* < 0.0001), lichen simplex chronicus (OR 1.44, *p* < 0.0001), dry skin (OR 1.53, *p* < 0.0001), and systemic lupus erythematosus (OR 2.15, *p* < 0.0001) (Figure 1). In contrast, black patients seen for itch were less likely to present with psoriasis (OR 0.52, *p* < 0.0001), contact dermatitis (OR 0.82, *p* < 0.0001), stasis dermatitis (OR 0.57, *p* < 0.0001), and scalp pruritus (OR 0.89, *p* < 0.0001) when compared to white patients.

**Figure 2:**
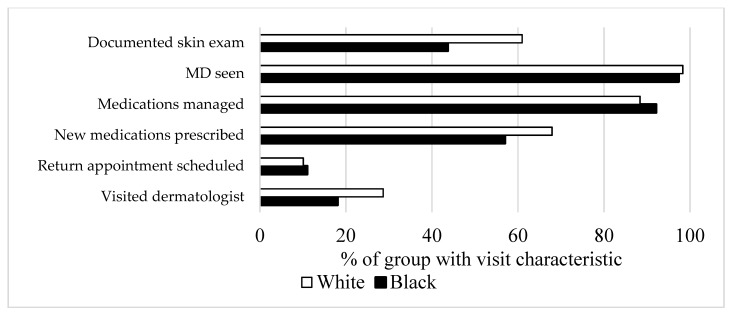
Visit characteristics for visits for itch nationally.

The differences in itch presentation can be explained by both physiologic variation and sociologic causes. To further explore the sociologic differences in pruritus presentation between black and white patients, we compared different visit characteristics in Figure 2 using NAMCS/NHAMCS datasets. Among the itch-related patients, more white patients had a history of a documented skin exam when compared to black patients (61% vs. 43%). Furthermore, white patients with itch symptoms were more likely to visit a dermatologist when compared to black patients with similar concerns, nationally (29% vs. 18%, OR 0.44, *p* = 0.028).

**Figure 3:**
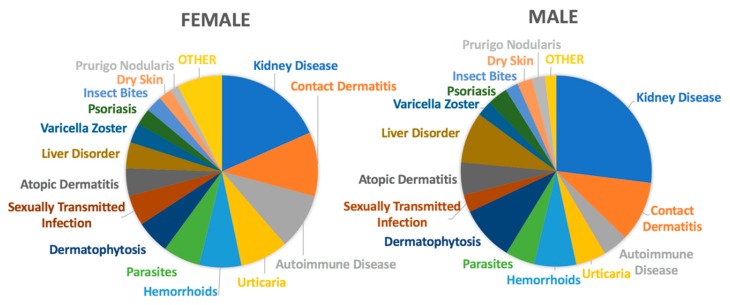
Gender differences in diagnosis.

**Figure 4:**
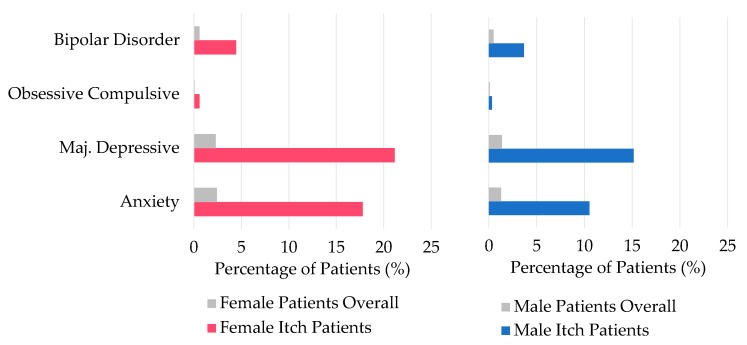
Comorbid psychiatric conditions in male and female pruritus patients compared to the general patient population at JHHS.

With regard to gender differences in the presentation of pruritus, females and males presented with different diagnoses for their itch symptoms at JHHS, as shown in Figure 3. Females reporting itch symptoms were more likely to be diagnosed with autoimmune conditions (OR 1.66, *p* < 0.0001), urticaria (OR 1.64, *p* < 0.0001), and sexually transmitted infections (OR 1.77, *p* < 0.0001) when compared to males reporting pruritus. However, women were less likely than men with itch to have kidney disease (OR 0.61, *p* < 0.0001), liver disease (OR 0.49, *p* < 0.0001), dermatophytes (0.59, *p* < 0.0001), or prurigo nodularis (0.61, *p* < 0.0001). Additionally, as shown in Figure 4, when investigating the prevalence of psychiatric comorbidities, both males and females suffering from pruritus were more likely to also be diagnosed with bipolar disorder, obsessive compulsive disorder, major depressive disorder, and generalized anxiety disorder when compared to the general patient population at JHHS (female: OR 7.1–9.6, *p* < 0.0001; male: OR 4.0–11.3, *p* < 0.0001). Of note, among the patients seen for pruritus, females are more likely than males to be diagnosed with these psychiatric comorbidities (OR 1.2–1.8, *p* < 0.0001).

## 4. Discussion

This study characterized racial and gender differences in the presentation of pruritus at John Hopkins Health System and compared the results to those seen nationally. Our findings indicate that a greater proportion of black patients are affected by pruritus compared to white patients, and these patients are more likely to be diagnosed with certain dermatologic conditions including prurigo nodularis, lichen planus, and atopic dermatitis. However, black patients reporting itch symptoms are less likely to visit a dermatologist when compared to their white counterparts. Female itch patients are more likely to be diagnosed with autoimmune conditions and psychiatric comorbidities when compared to male itch patients.

Despite the fact that patients with itch symptoms at JHHS were more likely to be female, black, and Hispanic or Latino, assessment of the national patient population partially confirmed these findings; a greater proportion of pruritus patients were black than non-pruritus patients, but there were not significant gender differences.

This study reports the increased risk in black itch patients for atopic dermatitis, dry skin, SLE, prurigo nodularis, lichen planus, and lichen simplex chronicus. While previous research has described these racial differences in atopic dermatitis and SLE, there is very little epidemiologic data on prurigo nodularis, lichen planus, and lichen simplex chronicus. Studies at our institution demonstrated that prurigo nodularis disproportionately affects black patients [8,9]. Our group also recently confirmed that PN disproportionately affects blacks and Asians in nationwide studies [10,11,12]. Our results indicating racial differences in the prevalence of certain dermatologic conditions can be attributed to the physiologic variation of skin between the races. For example, black skin has been found to have a lower pH, lower ceramide content within the stratum corneum, greater cohesion between keratinocytes and increased mast cells [13]. Knowledge of these differences and how they affect presentation of dermatologic symptoms such as pruritus can help guide treatment.

Despite the fact that black patients are more likely to report pruritus, we observed that even when adjusting for age, sex, socioeconomic status, and region, black itch patients were less likely to visit a dermatologist than white itch patients. Similarly, a study on the treatment of acne vulgaris demonstrated that black acne patients were less likely to visit a dermatologist than white acne patients [14]. Further research is necessary to validate these findings and better understand the cause for these disparities in the access and utilization of care.

In addition to the racial differences, there are several gender differences, as seen in Figure 3 and Figure 4, that may contribute to our understanding of pruritic symptoms. While autoimmune conditions, urticaria, and sexually transmitted infections are more likely to be associated with pruritus in females, liver and kidney disease, dermatophytosis, and prurigo nodularis are more likely to be diagnosed in males with pruritus. Many of these differences can be attributed to the epidemiology of the conditions themselves. Autoimmune conditions and urticaria both occur more commonly in women, perhaps because of differences in sex hormones and inflammation pathways, which are reflected in our findings [15]. Women are found to progress toward end-stage renal disease at a slower rate than men, thus explaining the gender discrepancy in pruritic presentation with kidney disease [16].

Although the exact pathophysiology of pruritus is not fully elucidated, the central neural mechanisms of itch have been suggested to be mediated by neuropeptides, especially endogenous opioids, and histamine [17,18]. Gupta et al. described the association between depression and elevated levels of corticotropin releasing factor, leading to increased central opiate levels and heightened perception of itch sensation [19]. Here, the increased risk of psychiatric comorbidities in all pruritus patients when compared to the general patient population underscores the strong central component of pruritus. Furthermore, women reporting pruritus symptoms are more likely than males to have comorbid psychiatric conditions. Studies of patients with chronic pruritus have shown that women have higher anxiety scores and levels of neuropathic and psychosomatic disease, which is in accordance with our findings [7]. Knowledge of the increased prevalence of psychiatric comorbidities in patients reporting pruritus will help providers better manage pruritic dermatologic disease.

Overall, even though itch is a common symptom in many diseases, there is little knowledge about the influence of gender and race in pruritus. Our study illustrates both gender and racial differences in the presentation of patients with pruritus symptoms. Future studies should investigate the causes of such differences and to address these disparities.

## Figures and Tables

**Table 1:** Characteristics of visits for itch at JHHS (2012–2018) and the United Sates (2005–2011).

Demographics	JHHS			NAMCS		
	ITCH (*n* = 18753)	NO ITCH (*n* = 4732084)		ITCH (*n* = 3778)	NO ITCH (*n* = 888990)	
	% visits	% visits	*p*-value	% visits	% visits	*p*-value
**Female**	66.73	54.26	0.01	58.97	56.42	0.61
**Male**	31.17	45.45	0.01	41.03	43.58	0.61
**Age**						
**Under 18 Years**				17.05	20.15	0.44
**18–24 Years**	11.25	11.64	0.90	6.80	6.88	0.97
**24–44 Years**	28.29	28.23	0.99	18.79	22.71	0.35
**45–64 Years**	33.65	31.04	0.57	18.79	29.10	0.02
**65–74 Years**	16.18	14.29	0.59	6.01	10.78	0.12
**75 Years and Older**	10.62	14.79	0.24	6.48	10.37	0.20
**Race**						
**White**	47.82	54.18	0.20	65.38	73.99	0.05
**Black**	37.49	19.01	<0.01	25.62	20.65	0.05
**Other**	14.86	16.24	0.71	9.00	5.36	0.11
**Ethnicity**						
**Hispanic or Latino**	5.81	2.70	0.05	17.50	15.02	0.49
**Not Hispanic or Latino**	90.16	95.30	0.05	82.50	84.98	0.49

## References

[1] Shive M., Linos E., Berger T., Wehner M., Chren M.-M. (2013). Itch as a patient-reported symptom in ambulatory care visits in the United States. J. Am. Acad. Dermatol..

[2] Ständer S., Steinhoff M., Schmelz M., Weisshaar E., Metze D., Luger T. (2003). Neurophysiology of Pruritus: Cutaneous Elicitation of Itch. Arch. Dermatol..

[3] Lee C., Chuang H., Shih C., Jong S., Chang C., Yu H. (2006). Transepidermal water loss, serum IgE and b-endorphin as important and independent biological markers for development of itch intensity in atopic dermatitis. Br. J. Dermatol..

[4] Szepietowski J.C., Reich A. (2016). Pruritus in psoriasis: An update. Eur. J. Pain.

[5] Mrowietz U., Chouela E.N., Mallbris L., Stefanidis D., Marino V., Pedersen R., Boggs R.L. (2015). Pruritus and quality of life in moderate-to-severe plaque psoriasis: Post hoc explorative analysis from the PRISTINE study. Eur. Acad. Dermatol. Venereol..

[6] Patel S., Vasavda C., Ho B., Meixiong J., Dong X., Kwatra S. (2019). Cholestatic pruritus: Emerging mechanisms and therapeutics. J. Am. Acad. Dermatol..

[7] Ständer S., Stumpf A., Osada N., Wilp S., Chatzigeorgakidis E., Pfleiderer B. (2013). Gender differences in chronic pruritus: Women present different morbidity, more scratch lesions and higher burden. Br. J. Dermatol..

[8] Boozalis E., Tang O., Patel S., Semenov Y.R., Pereira M.P., Stander S., Kang S., Kwatra S.G. (2018). Ethnic differences and comorbidities of 909 prurigo nodularis patients. J. Am. Acad. Dermatol..

[9] Bender A.M., Tang O., Khanna R., Ständer S., Kang S., Kwatra S.G. (2019). Racial differences in dermatological conditions associated with Human Immunodeficiency Virus: A cross-sectional study of 4,679 patients in an urban tertiary care center. J. Am. Acad. Dermatol..

[10] Whang K.A., Kang S., Kwatra S.G. (2019). Inpatient Burden of Prurigo Nodularis in the United States. Medicines.

[11] Huang A.H., Canner J.K., Kang S., Kwatra S.G. (2019). Analysis of real-world treatment patterns in patients with prurigo nodularis. J. Am. Acad. Dermatol..

[12] Huang A.H., Canner J.K., Khanna R., Kang S., Kwatra S.G. (2019). Real-world prevalence of prurigo nodularis and burden of associated diseases. J. Investig. Dermatol..

[13] Czerkasij V. (2013). Skin of color. Nurse Pract..

[14] Rogers A.T., Semenov Y.R., Kwatra S.G., Okoye G.A. (2018). Racial disparities in the management of acne: Evidence from the National Ambulatory Medical Care Survey, 2005–2014. J. Dermatolog. Treat..

[15] Fairweather D., Frisancho-Kiss S., Rose N.R. (2008). Sex Differences in Autoimmune Disease from a Pathological Perspective. Am. J. Pathol..

[16] Cobo G., Hecking M., Port F.K., Exner I., Lindholm B., Stenvinkel P., Carrero J.J. (2016). Sex and gender differences in chronic kidney disease: progression to end-stage renal disease and haemodialysis. Clin. Sci..

[17] Lee H.G., Stull C., Yosipovitch G. (2017). Psychiatric disorders and pruritus. Clin. Dermatol..

[18] Jafferany M., Davari M.E. (2019). Itch and psyche: Psychiatric aspects of pruritus. Int. J. Dermatol..

[19] Gupta M., Gupta A., Schork N., Ellis C. (1994). Depression modulates pruritus perception: A study of pruritus in psoriasis, atopic dermatitis, and chronic idiopathic urticaria. Psychosom. Med..

